# Transformative connections: from collaboration to impact in healthcare quality and safety

**DOI:** 10.31744/einstein_journal/2026Suppl_4ED0001

**Published:** 2026-07-20

**Authors:** Paula Tuma

**Affiliations:** 1 Department of Quality, Safety and Healthcare-Associated Infection Prevention and Control Hospital Israelita Albert Einstein São Paulo SP Brazil Department of Quality, Safety and Healthcare-Associated Infection Prevention and Control, Hospital Israelita Albert Einstein, São Paulo, SP, Brazil.

Improvements in healthcare quality and safety have never been the result of isolated efforts by individuals or institutions. They arise from the ability to connect people, experiences, knowledge, and evidence around a common purpose: to provide care that is increasingly safe, effective, person-centered, equitable, and sustainable. In the context of increasingly complex healthcare systems, quality is no longer merely a set of specific initiatives or projects but has become a central element of organizational strategy, guiding decisions, priorities, and learning processes at all levels of the system.^(1,2)^ This vision recognizes that creating value for patients, healthcare professionals, and communities depends on organizations’ ability to continuously learn, adapt, and respond resiliently to ever-changing needs.^(2)^

It is in this spirit that the journal **einstein** (São Paulo) presents this special issue, featuring the scientific abstracts from the 11th Latin American Forum on Quality and Safety in Healthcare, organized by *Hospital Israelita Albert Einstein* in partnership with the Institute for Healthcare Improvement (IHI). Under the theme “A Focus on the Patient: Connections That Transform,” the Forum reaffirms the conviction that has guided its journey since its first edition: the transformation of healthcare systems occurs when different perspectives come together to learn, co-create, and innovate.

The theme of this issue aligns with the contemporary understanding of quality in healthcare: that sustainable outcomes are achieved when different stakeholders share a sense of purpose, engage in collective learning, and develop solutions collaboratively.^(2,3)^ More than simply connecting institutions, it is about connecting perspectives, experiences, and knowledge capable of transforming entire healthcare systems. The fundamental question guiding this transformation remains the same question that has inspired generations of healthcare professionals: how does every decision, innovation, or change contribute to improving the health of the people our healthcare systems serve?^(4)^

Over the course of more than a decade, the Forum has established itself as one of the leading platforms for knowledge exchange on quality and safety in Latin America. By bringing together healthcare professionals, managers, researchers, patients, public policymakers, and leaders of healthcare organizations, the event strengthens a community committed to the continuous improvement of care and the building of more resilient healthcare systems. This ability to bring together professionals from diverse fields and contexts also reflects one of the most important attributes of learning organizations: creating spaces where dialogue, curiosity, and the exchange of experiences foster innovation, the development of local capacities, and the dissemination of successful practices.^(2)^

It is important that the connections made during the Forum not be limited to the event itself. Their true potential lies in their ability to generate shared knowledge, foster new collaborations, and disseminate practices that can be adapted and replicated in different contexts. The dissemination of knowledge is one of the most powerful mechanisms for accelerating large-scale improvements. By sharing experiences, successes, and challenges, organizations contribute to building healthcare systems that are more responsive, resilient, and capable of meeting the needs of patients and communities in a continuous, reliable, and sustainable manner.^(2)^ It is in this sense that this collection of abstracts plays a role that goes beyond scientific documentation: it provides a living snapshot of the initiatives currently being developed in hospitals, healthcare services, universities, and public and private organizations throughout Latin America.

In this issue of journal **einstein** (São Paulo), we present the selected scientific abstracts from the Forum, reflecting the maturity of initiatives in the region. The abstracts highlight the diversity of challenges faced by contemporary healthcare systems. Although they address different contexts, they all share a common element: a commitment to creating value for patients, healthcare professionals, and society. This diversity highlights that quality improvement is not the sole responsibility of specialists or specific departments, but rather a collective effort involving leadership, organizational culture, continuous learning, and the active participation of all stakeholders in the healthcare system.^(2,5,6)^The development of organizational capacities to simultaneously improve the quality of care, the health of populations, and the sustainability of healthcare systems depends on leaders capable of mobilizing people around shared and enduring goals.^(5,6)^

We hope this special issue will inspire new questions, foster partnerships among institutions, strengthen collaborative networks, and encourage researchers and professionals to transform local experiences into shared knowledge. In a field where challenges are increasingly complex, no organization can evolve on its own. Collective learning remains one of the most powerful drivers of innovation in healthcare.^(2)^ Similarly, the leadership required to address these challenges demands steadfast purpose, a commitment to people’s well-being, the ability to listen, and the creation of environments that foster trust, collaboration, and a safe space for learning.^(6)^ These are the elements that underpin the lasting transformation of healthcare systems and expand their capacity to generate better outcomes for patients, healthcare professionals, and society.^(3)^

May the abstracts gathered here broaden the dialogue between science and practice and help ensure that the connections forged during the 11th Latin American Forum on Quality and Safety in Healthcare continue to have an impact far beyond the event itself. After all, when knowledge, collaboration, purpose, and a commitment to people come together, the conditions are created for improvement to cease being a collection of isolated initiatives and become an integral part of the culture, leadership, and strategy of healthcare systems. It is in this convergence that connections truly bring about transformation.

Enjoy your reading!
